# Muscle morphological changes and enhanced sprint running performance: A 1‐year observational study of well‐trained sprinters

**DOI:** 10.1002/ejsc.12155

**Published:** 2024-06-21

**Authors:** Raki Kawama, Katsuki Takahashi, Hironoshin Tozawa, Takafumi Obata, Norifumi Fujii, Aya Arai, Tatsuya Hojo, Taku Wakahara

**Affiliations:** ^1^ Faculty of Health and Sports Science Doshisha University Kyotanabe‐shi Kyoto Japan; ^2^ Organization for Research Initiatives and Development Tokyo Japan; ^3^ Graduate School of Health and Sports Science Doshisha University Kyotanabe‐shi Kyoto Japan; ^4^ Human Performance Laboratory Waseda University Saitama Japan

**Keywords:** daily training, hypertrophy, maximal velocity phase, thigh, trunk

## Abstract

Numerous cross‐sectional studies have attempted to identify the muscle morphology required to achieve high sprint velocity. Our longitudinal study addressed an unanswered question of cross‐sectional studies: whether hypertrophy of the individual trunk and thigh muscles induced by daily training (e.g., sprint, jump, and resistance training) is linked to an improvement in sprint performance within well‐trained sprinters. Twenty‐three collegiate male sprinters (100‐m best time of 11.36 ± 0.44 s) completed their daily training for 1 year without our intervention. Before and after the observation period, the sprint velocities at 0–100 m, 0–10 m, and 50–60 m intervals were measured using timing gates. The volumes of 14 trunk and thigh muscles were measured using magnetic resonance imaging. Muscle volumes were normalized to the participants' body mass at each time point. Sprint velocities increased at the 0–100 m (*p* < 0.001), 0–10 m (*p* = 0.019), and 50–60 m (*p* = 0.018) intervals after the observation period. The relative volumes of the tensor fasciae latae, sartorius, biceps femoris long head, biceps femoris short head, semitendinosus, and iliacus were increased (all *p* < 0.050). Among the hypertrophied muscles, only the change in the relative volume of the semitendinosus was positively correlated with the change in sprint velocity at the 50–60 m interval (*p* = 0.018 and *ρ* = 0.591). These findings suggest that semitendinosus hypertrophy seems to be associated with sprint performance improvement within well‐trained sprinters during the maximal velocity phase.

## Highlights

- Over the past few decades, numerous cross‐sectional studies have attempted to identify the muscle morphology required to achieve high sprint velocity, but the question remains as to whether hypertrophy of the individual trunk and thigh muscles is linked to an improvement in sprint performance within well‐trained sprinters.
- After 1‐year daily training in well‐trained sprinters, the tensor fasciae latae, sartorius, biceps femoris long head, biceps femoris short head, semitendinosus, and iliacus were hypertrophied.
- Among the hypertrophied muscles, only semitendinosus hypertrophy was associated with sprint performance improvement within well‐trained sprinters during the maximal velocity phase of the 100‐m sprint.

## 1 INTRODUCTION

Sprint running is the fastest mode of human locomotion. Improving the sprint performance (accelerating quickly and achieving and maintaining a high maximal running velocity) is a key challenge in various sporting events that require sprint ability. Theoretically, to bring the body forward faster during a sprint, humans must generate muscular power (the rate of mechanical work (Cormie et al., 2011)) in the lower body (Chelly et al., 2001). The muscular power is primarily determined by muscle size, particularly muscle volume (O’Brien et al., 2009). Thus, it can be expected that hypertrophy of the muscles crossing the lower limb joints is advantageous for achieving high sprint velocities. However, the hypertrophy of muscles with a low contribution to sprint running entails an increase in body mass, which can impair the sprint velocity (Ropret et al., 1998). From these perspectives, it is crucial to better understand which muscles should be hypertrophied to achieve high sprint velocities.

Over the past few decades, numerous cross‐sectional studies have attempted to identify the muscle morphology required to achieve high sprint velocity (Ema et al., 2018; Miller et al., 2021, 2022; Sugisaki et al., 2018; Takahashi et al., 2021a; Tottori et al., 2021). For example, a high sprint performance (shorter sprint time and/or higher sprint velocity) was observed in sprinters with large muscle sizes (relative to body mass) of the sartorius (SAR (Miller et al., 2022)), rectus femoris (RF (Ema et al., 2018)), semitendinosus (ST (Takahashi et al., 2021a)), gluteus maximus (*G*_max_ (Miller et al., 2021)), psoas major (PM (Sugisaki et al., 2018)), adductor (ADD) magnus (Miller et al., 2022), thigh ADDs (Nuell et al., 2019), and hamstrings (Nuell et al., 2019; Sugisaki et al., 2018). These cross‐sectional findings suggest that the interindividual variability in the sizes of specific trunk and thigh muscles is responsible for the interindividual differences in sprint performance. Meanwhile, these cross‐sectional studies have left an unanswered question: whether hypertrophy of the individual trunk and thigh muscles induced by daily training (e.g., sprint, jump, and resistance training) is linked to an improvement in sprint performance within well‐trained sprinters. Sprint performance depends on multiple factors including morphological, neurophysiological, histochemical, and biomechanical factors (Majumdar et al., 2011). Additionally, these relevant factors were shown to change through resistance and/or technical training (Häkkinen et al., 1998; Mendiguchia et al., 2022). Thus, it is possible that the hypertrophy of the individual trunk and thigh muscles induced by daily training is not necessarily associated with an improvement in sprint performance within well‐trained sprinters. Addressing the questions of cross‐sectional studies could be informative in developing effective training strategies for sprint performance improvement.

To the best of our knowledge, only one longitudinal study has investigated changes in the relative volumes of lower limb muscle groups (e.g., quadriceps femoris and hamstrings) and their associations with changes in sprint performance (Nuell et al., 2019). However, these muscle groups were not separated into the individual muscles in the previous study (Nuell et al., 2019), although the individual quadriceps femoris and hamstring muscles were reported to exhibit different hypertrophic responses to resistance training (Bourne et al., 2017; Ema et al., 2013). Moreover, the previous study (Nuell et al., 2019) did not assess volumes of SAR, *G*_max_, and PM, which were shown to be significantly correlated with the sprint performance in cross‐sectional studies (Miller et al., 2021, 2022; Sugisaki et al., 2018; Tottori et al., 2021). Thus, the present study aimed to investigate whether the morphological changes of 14 individual trunk and thigh muscles induced by 1‐year daily training are associated with an improvement in the sprint performance within well‐trained sprinters.

## 2 MATERIALS AND METHODS

### 2.1 Participants

Twenty‐nine well‐trained male collegiate sprinters were recruited for this study. For these participants, the experimental data were collected from November 2021 to November 2022. During the 1‐year experimental period, six athletes dropped out of this study due to severe musculoskeletal injuries, COVID‐19 infection, and withdrawal from the university's athletic club. Thus, a total of 23 athletes completed the present experiment. All participants met the following three criteria: (1) performing high‐velocity, short‐distance sprint running as a part of their daily training; (2) having ≥3 years of experience in training for improving sprint performance; and (3) having no history of musculoskeletal injuries or neurological disorders of the right trunk and thigh within the past 6 months. All participants completed an informed consent form after a careful explanation of the purpose, procedures, and potential risks of this study. The present study was approved by the Research Ethics Committee of the authors' institution (no. 21035) and conducted in accordance with the Declaration of Helsinki.

### 2.2 Experimental design

**FIGURE 1:**
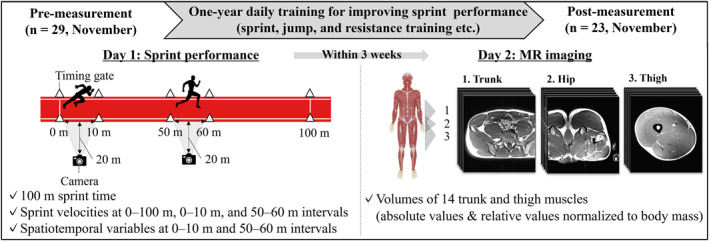
Overview of the experimental design and measured variables.

The athletes (*n* = 23) completed daily training between November 2021 and November 2022 (Figure 1). During the experimental period, they never failed to participate in the daily training for 5 consecutive days. Because this study was designed as an observational study, we did not interfere with the athletes' training schedules or processes throughout the 1‐year observation period. The athletes participated in measurement sessions of sprint performance and muscle size across 2 days before and after the observation period, respectively. On the first day, 100‐m sprint tests were performed on an outdoor synthetic running track to assess the mean sprint velocities and the spatiotemporal variables (step frequency, step length, flight time, stance time, flight distance, and stance distance). On the second day, magnetic resonance (MR) images of the trunk, hip, and thigh were captured to calculate the volumes of the trunk and thigh muscles. The interval between the two measurement sessions ranged from 1 to 3 weeks among the athletes.

### 2.3 Sprint performance measurement

To assess the sprint times at 0–100, 0–10, and 50–60 m intervals, five pairs of timing gates (Witty Gate, Microgate) were positioned at 0, 10, 50, 60, and 100 m from the starting line. The timing gates were set at a height of 1 m, which approximately matched the height of the participant's hip joints, to minimize the measurement errors of sprint time (Yeadon et al., 1999). To capture the participants' sprints at the 0–10 and 50–60 m intervals, two cameras were placed at 5 m (EX‐100PRO, CASIO, Japan, frame rate: 240 fps, exposure time: 1/2000s) and 55 m (HAS‐U2, DETECT, Japan, frame rate: 250 fps, exposure time: 1/3000 s) from the starting line on the right side of the running lane, respectively. The reference markers were placed at 0, 10, 50, and 60 m from the starting line in the middle of the running lane for spatial calibration of the captured video. The two cameras were placed at 20 m perpendicularly from the midpoint of the lane. Special care was taken to ensure that the setup of the timing gates and cameras were the same before and after the observation period. Based on a previous report (Ae et al., 1992), we defined the 0–10 m and 50–60 m intervals as the initial acceleration and maximum velocity phases, respectively. To evaluate the wind velocity in the direction of sprints, a digital anemometer (866B Pro Anemometer, Kethvoz) was positioned 50 m from the starting line. The wind velocity was measured just after the athletes started sprinting. The tailwind and headwind were presented as plus and minus, respectively. The temperatures at the experimental site were recorded using the official information published by the Japan Meteorological Agency. The sprint performance measurements were not implemented on days with low temperatures (less than 10°C) and/or rain.

After a warm‐up consisting of light jogging, dynamic stretching exercises, and short sprints, the athletes performed two 100‐m sprints at maximal effort with their sprint spikes. Starting blocks were used for the 100‐m sprints. The setup of the starting blocks (block obliquity and distance between the starting line and blocks) was carefully matched before and after the observation period. The athletes started sprints in their arbitrary timing after the investigator carefully confirmed that the wind velocity was less than 2.0 m/s. A rest period of 40 min was allowed between two trials. A trial with the shorter 100‐m sprint time was used for subsequent analyses.

The mean velocity of the 100‐m sprint was calculated by dividing the running distance (100 m) by the time of the 100‐m sprint. The mean sprint velocities at the 0–10 m and 50–60 m intervals were calculated by dividing the interval distance (10 m) by the sprint time at each interval. Considering the potential effects of wind velocity on sprint performance, the velocities of 100‐m sprints were corrected using an equation from a previous study (Moinat et al., 2018). The spatiotemporal variables for the right leg were calculated from sprints captured at the 0–10 m and 50–60 m intervals based on the calculation processes of a previous study (Takahashi et al., 2021a). The horizontal distance in pixels between the reference markers was converted to the actual distance (10 m) at each interval using Image J software (National Institute of Health, USA). A running cycle was defined as the period from the touchdown of the right foot to the next touchdown of the same foot. The stance phase of the right leg was defined as the part of a running cycle from the touchdown of the right foot to right toe‐off, whereas the flight phase was defined as that from the right toe‐off to the touchdown of the left foot. The stance and flight times were calculated by dividing each frame count during the stance or flight phase by the frame rate of the cameras. The sum of the stance and flight times was defined as the step time, and the inverse of the step time was defined as the step frequency. The stance and flight distances were defined as the horizontal distances traveled by the anterior‐posterior center of the pelvis during the stance and flight phases, respectively. The horizontal distance during the stance and flight phases was calculated by digitizing the anterior‐posterior center of the pelvis at touchdown of the right‐foot, right toe‐off, and touchdown of the left foot using Image J software. The sum of the stance and flight distances was presented as the step length. At 0–10 m interval, the spatiotemporal variables were calculated twice for the third cycle of the right leg from the starting line because the sprint velocity (the product of step frequency and step length) in the third cycle was more closely related to the mean sprint velocity at the 0–10 m interval compared to that in the first and second cycles in our preliminary analysis. The mean value of the two calculated values was used for subsequent analysis. At 50–60 m interval, spatiotemporal variables were calculated twice for the first and second cycles from the 50‐m point, respectively. The mean value of the four calculated values was used for subsequent analyses. To test the intra‐rater reliability of the analysis, spatiotemporal variables were analyzed twice for 10 participants. At the 0–10 m interval, CV and ICC (1, 2) ranged from 0.8% to 4.2% and 0.92 to 0.99, respectively. At the 50–60 m interval, CV and ICC (1, 2) ranged from 0.6% to 2.7% and 0.81 to 0.99, respectively.

### 2.4 MR imaging measurement

T1‐weighted MR images of the right trunk, hip, and thigh were obtained using body array and spine coils (Body 18 and CP Spine Array Coil, Siemens Healthineers, Germany [field of view, 300 × 300–350 × 350; matrix, 256 × 256–384 × 384; slice thickness, 5 mm; pixel size, 1.17 × 1.17–1.37 × 1.37 mm; repetition time, 420–600 ms; echo time, 8.6–14.0 ms; gap, 5 mm; and the number of slices, 18 × 2 to 4 blocks]). The athletes were required to refrain from strenuous exercise and practice for 1 day before the MR imaging. Prior to scanning, the athletes lay supine for at least 20 min to minimize the effect of fluid shift due to postural changes on the cross‐sectional areas of the trunk and thigh muscles. To scan images of the hip, the athletes lay prone with their legs extended and muscles fully relaxed in a 3.0 T magnet bore (MAGNETOM Skyra, Siemens Healthineers). Cushions were placed on (or under) some body parts to avoid contact between the coils and the examined muscles. To obtain images of the trunk and thigh, the athletes lay supine with their legs extended. The right foot was placed into a handmade pad to prevent hip joint rotation. During the scanning of the hip and trunk, the athletes were asked to hold their breath for 20 s to prevent the potential influence of motion artifacts due to respiration. The MR images of the trunk, hip, and thigh were taken to cover the whole part of all examined muscles.

In the MR images, the outline of each muscle was semiautomatically traced using the image analysis software (SASHIMI segmentation, Bartbols). The anatomical cross‐sectional areas were analyzed from the origins to insertions for the following 14 trunk and thigh muscles: (1) tensor fasciae latae (TFL), (2) SAR, (3) gracilis (Gra), (4) RF, (5) vastus lateralis and intermedius (VLVI), (6) vastus medialis (VM), (7) thigh ADDs, (8) biceps femoris long head (BFlh), (9) biceps femoris short head (BFsh), (10) ST, (11) semimembranosus (SM), (12) *G*_max_, (13) PM, and (14) iliacus (IL). When errors and overlaps occurred between adjacent muscle cross‐sections, the outline of the muscle was manually traced. It was difficult to identify the outlines between the adductor magnus, longus, brevis, and pectineus along the thigh; thus, these four muscles were pooled as the thigh ADDs (except for Gra). Additionally, the border of VL and VI could not be clearly identified, especially at the proximal part of the thigh. Hence, the two muscles were pooled as VLVI. In the traced area, the anatomical cross‐sectional area of each muscle was calculated as the product of the pixel size and number of pixels using MATLAB 2021b (MathWorks Inc., USA). The total volume of the individual muscles was determined by summing the anatomical cross‐sectional areas of each image times 1 cm (the sum of the slice thickness and interslice gap). The muscle volume was normalized to body mass as the relative muscle volume (cm^3^ ·kg^−1^). To test the intra‐rater reliability of the muscle volume analysis, the absolute volumes of the 14 examined muscles in seven athletes were measured twice. The CVs and ICCs (1, 2) ranged from 0.9% to 4.0% and 0.81 to 0.99, respectively.

### 2.5 Daily training for improving the sprint performance

During the observation period, we carefully recorded the content of the athletes' daily training (e.g., the type of training, intensity, and average weekly volume [total distance and total number of steps and repetitions]) from the training diary of the university's athletics club (Supporting Information S1). The daily training was divided into preparatory (November to February) and competition (March to October) periods. During both periods, the athletes trained 5 days per week. At the beginning of each training day, they performed light jogging and dynamic stretching. The weekly training consisted of three sessions of flat terrain sprint running, one session of uphill sprint running, five sessions of sprint‐technique drills, two sessions of horizontal jumps, two sessions of vertical jumps, two sessions of repetition maximum‐based exercises, and five sessions of body mass‐based exercises for the trunk and thigh muscles.

### 2.6 Statistical analysis

The Shapiro–Wilk normality test was used to assess the distribution of the sprint performance variables (100‐m sprint time; sprint velocities at the 0–100 m, 0–10 m, and 50–60 m intervals; and spatiotemporal variables at the 0–10 m and 50–60 m intervals) and absolute volumes of the 14 trunk and thigh muscles. As a result, some data were non‐Gaussian (*p* = 0.001–0.999). Thus, the Wilcoxon signed‐rank test was performed to identify differences in the measured variables before and after the 1‐year observation period. The Spearman's rank correlation test was used to investigate the correlations between the changes in the sprint velocities at the 0–100 m, 0–10 m, and 50–60 m intervals and the changes in the relative volumes of the significantly hypertrophied trunk and thigh muscles. When significant correlations were found, the Spearman's rank correlation test was performed to examine the correlations between changes in the relative muscle volume and changes in the spatiotemporal variables at the corresponding interval. The significance level was set at *p* < 0.05. For multiple correlation analyses, the *p*‐value was corrected based on the number of analyzed muscles using the Benjamini and Hochberg method (Glickman et al., 2014) at a false discovery rate of <0.05. The effect size (*r*) was calculated for all the tests, except for the Spearman's rank correlation test. The data are presented as median values (interquartile ranges). All statistical analyses were performed using a statistical software package (IBM SPSS Statistics, version 28.0, IBM Corporation, Armonk, USA) and a web application (Langtest, Atsushi Mizumoto, Kansai University, Japan [Mizumoto and Plonsky, 2016]).

## 3 RESULTS

### 3.1 Participant's information at the beginning of the experiment

The age, body height, and mass of 23 athletes were 19.7 ± 1.0 years, 173.5 ± 6.0 cm, and body mass 66.1 ± 5.6 kg, respectively. They engaged in 100–400 m sprints (*n* = 16), long and triple jumps (*n* = 4), and decathlon (*n* = 3). Their personal best records for the 100‐m race ranged from 10.50 to 12.10 s (11.36 ± 0.44 s) at the beginning of the experiment. They had ≥4 years (7.9 ± 1.8 years) of experience in their athletic disciplines.

### 3.2 Changes in the anthropometric variables

Before and after the 1‐year observation period, the body height did not significantly change (*p* = 0.115, *r* = 0.23, and from 172.5 cm [169.3–179.1 cm] to 172.8 cm [169.2–178.6 cm]). The body mass also did not significantly change (*p* = 0.622, *r* = 0.07, and from 66.0 kg [61.3–71.0 kg] to 67.0 kg [62.9–70.1 kg]).

### 3.3 Differences in the environmental variables and changes in the sprint performance

The temperatures at the time of sprint performance measurement did not significantly differ (*p* = 0.080, *r* = 0.26, and from 16.0°C [14.9–18.0°C] to 16.4°C [16.0–18.2°C]) before and after the observation period. The wind velocity was significantly higher after the observation period (*p* = 0.002, *r* = 0.45, and from +0.60 m/s [+0.30 to +1.15 m/s] to +1.40 m/s [+1.10 to +1.50 m/s]).

During the observation period, 17 athletes participated in an official 100‐m race and seven of them broke their own personal best records. The personal best record for the 100‐m race for all athletes was 11.40 s (10.94–11.73 s) and 11.34 s (10.91–11.73 s) before and after the observation period, respectively. The 100‐m sprint time was significantly shortened after the observation period (*p* < 0.001 and *r* = 0.50; Table 1). There were significant increases in sprint velocities at the 0–100 m (*p* < 0.001 and *r* = 0.49), 0–10 m (*p* = 0.019 and *r* = 0.35), and 50–60 m (*p* = 0.018 and *r* = 0.35) intervals after the observation period. The sprint velocity with correction for wind velocity at 0–100 m interval also significantly increased after the observation period (*p* = 0.002 and *r* = 0.46).

**TABLE 1:** Changes in the sprint performance variables after the 1‐year observation period.

Interval	Sprint performance variable	Before		After		% Change	
100 m	**Time (s)**	**11.69**	11.47–12.00	**11.49**	10.99–11.76	**−1.5**	−4.2 to −0.1**
	**Velocity** (m∙s^−1^)	**8.55**	8.34–8.72	**8.70**	8.51–9.10	**1.5**	0.1–4.2**
	**Corrected velocity** (m∙s^−1^)	**8.49**	8.31–8.72	**8.64**	8.46–9.03	**1.6**	0.3–3.7**
0–10 m	**Velocity** (m∙s^−1^)	**5.38**	5.29–5.46	**5.43**	5.31–5.62	**1.2**	−0.8–3.4*
	**Step frequency (Hz)**	**4.615**	4.330–4.710	**4.661**	4.461–4.831	**2.9**	0.5–7.2**
	Step length (m)	1.527	1.448–1.643	1.519	1.479–1.593	−1.8	−5.0–3.3
	**Flight time (s)**	**0.094**	0.081–0.106	**0.083**	0.078–0.095	**−13.8**	−20.9–1.43*
	Stance time (s)	0.125	0.118–0.133	0.127	0.115–1.136	−1.5	−7.2–9.0
	Flight distance (m)	0.662	0.559–0.743	0.630	0.595–0.677	−5.9	−17.3 to 2.0
	Stance distance (m)	0.854	0.822–0.914	0.866	0.818–0.957	1.3	−3.5–8.4
50–60 m	**Velocity** (m∙s^−1^)	**9.52**	9.22–9.80	**9.71**	9.43–10.00	**0.9**	0.0–4.3*
	Step frequency (Hz)	4.609	4.446–4.752	4.594	4.477–4.763	0.6	−1.9–3.6
	Step length (m)	2.107	1.993–2.192	2.130	2.041–2.189	1.5	−1.0–3.2
	Flight time (s)	0.120	0.116–0.130	0.123	0.118–0.131	2.2	−2.7–5.7
	**Stance time (s)**	**0.095**	0.093–0.098	**0.093**	0.088–0.095	**−3.3**	−7.6 to 0.5*
	**Flight distance (m)**	**1.139**	1.035–1.217	**1.182**	1.126–1.234	**5.3**	−0.6–6.8*
	Stance distance (m)	0.950	0.916–0.977	0.935	0.902–0.984	−0.7	−5.9 to 2.0

*Note*: The data are presented as median values (interquartile range).

Significant differences before and after the 1‐year observation period: *p < 0.05 and **p < 0.01. Measured variables with significant changes are emboldened.

At the 0–10 m interval, the step frequency significantly increased (*p* = 0.006 and *r* = 0.41), whereas the flight time significantly shortened (*p* = 0.030 and *r* = 0.32) after the observation period. The other spatiotemporal variables did not significantly change at the 0–10 m interval before and after the observation period (*p* = 0.247–0.891 and *r* = 0.02–0.25). At the 50–60 m interval, the stance time was significantly shortened (*p* = 0.015 and *r* = 0.47), whereas the flight distance significantly increased (*p* = 0.013 and *r* = 0.37) after the observation period. There were no significant changes in the other spatiotemporal variables at the 50–60 m interval before and after the observation period (*p* = 0.066–0.463 and *r* = 0.11–0.27). To represent the typical values of the data set, the changes in sprint performance variables are also presented as mean ± standard deviation in Supporting Information S2.

### 3.4 Changes in the absolute and relative muscle volumes

**FIGURE 2:**
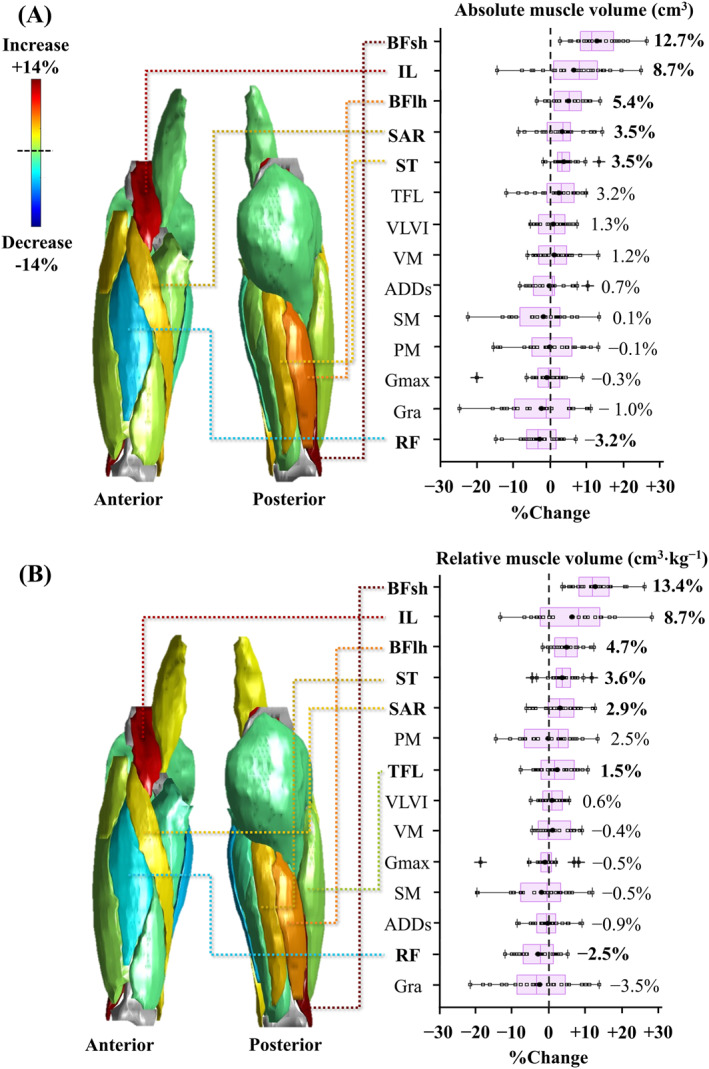
Boxplots of percent changes in (A) absolute and (B) relative volumes of 14 trunk and thigh muscles before and after the 1‐year observation period. The median values of the percent changes in the measured variables are presented on the right side of each boxplot. Measured variables with significant changes in the absolute and/or relative volumes are emboldened. +, outlier value; ●, the group mean value; ADDs, adductors; BFlh, biceps femoris long head; BFsh, biceps femoris short head; *G*_max_, gluteus maximus; Gra, gracilis; IL, iliacus; PM, psoas major; RF, rectus femoris; SAR, sartorius; SM, semimembranosus; ST, semitendinosus; TFL, Tensor fasciae latae; VLVI, vastus lateralis and intermedius; VM, vastus medialis.

The absolute muscle volumes of SAR (*p* = 0.015 and *r* = 0.36), BFlh (*p* < 0.001 and *r* = 0.61), BFsh (*p* < 0.001 and *r* = 0.76), ST (*p* < 0.001 and *r* = 0.61), and IL (*p* = 0.004 and *r* = 0.43) significantly increased, whereas that of RF (*p* = 0.020 and *r* = 0.34) significantly decreased after the observation period (Figure 2, Supporting Information S3). There were no significant changes in the absolute volumes of the other examined muscles before and after the observation period (*p* = 0.070–0.988 and *r* = 0.01–0.76). The relative muscle volumes of TFL (*p* = 0.048 and *r* = 0.29), SAR (*p* = 0.018 and *r* = 0.35), BFlh (*p* < 0.001 and *r* = 0.67), BFsh (*p* < 0.001 and *r* = 0.76), ST (*p* < 0.001and *r* = 0.52), and IL (*p* = 0.006 and *r* = 0.41) increased significantly after the observation period. Meanwhile, the relative volume of RF (*p* = 0.008 and *r* = 0.39) significantly decreased after the observation period. In the other examined muscles, no significant changes were observed in the relative muscle volumes before and after the observation period (*p* = 0.151–0.917 and *r* = 0.02–0.21).

### 3.5 Relationships between changes in the relative muscle volumes and sprint performance

**FIGURE 3:**
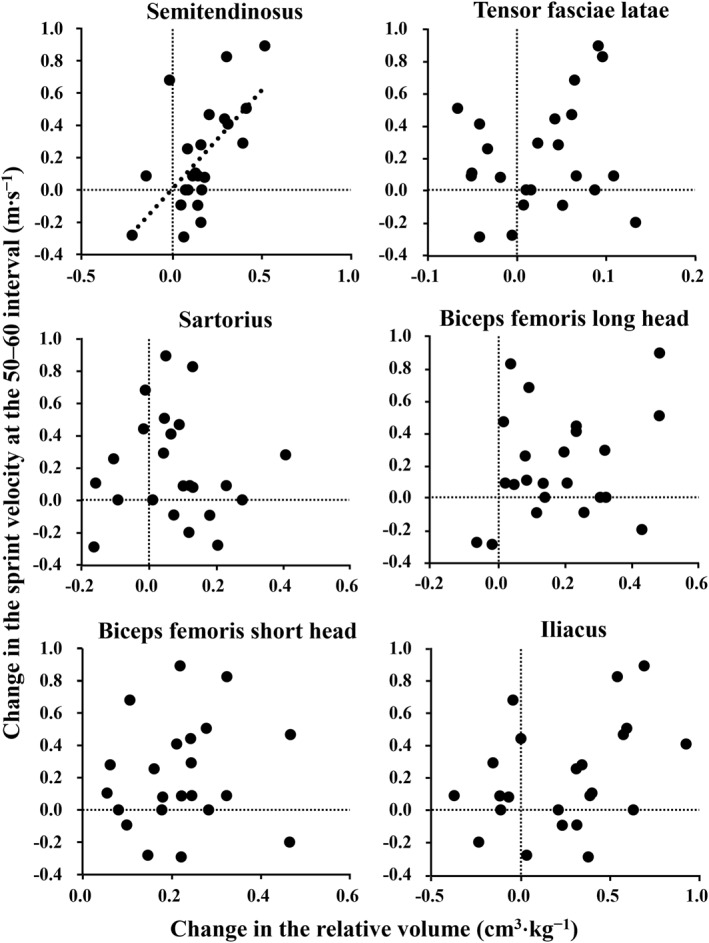
Scatter plots of the relationship between the changes in the relative volumes of the hypertrophied muscles and the change in sprint velocity at the 50–60 m interval after the 1‐year observation period.

The change in the relative volume of ST was significantly positively correlated with the change in the sprint velocity at the 50–60 m interval (*p* = 0.018 and *ρ* = 0.591; Figure 3, Supporting Information S4). However, the change in the relative ST volume was not significantly correlated with the changes in any spatiotemporal variables at the 50–60 m interval (*p* = 0.791–0.926 and *ρ* = −0.219 to 0.213). The changes in the relative volumes of the other hypertrophied muscles (TFL, SAR, BFlh, BFsh, or IL) were not significantly correlated with the changes in the sprint velocity at the 50–60 m interval (*p* = 0.270–0.546 and *ρ* = −0.159–0.361). In any hypertrophied muscles, changes in the relative volumes were not significantly correlated with the changes in sprint velocity at the 0–100 m (*p* = 0.234–0.545 and *ρ* = −0.133–0.433) or 0–10 m (*p* = 0.078–0.918 and *ρ* = 0.023–0.510) intervals. Similarly, the changes in the relative volumes of the hypertrophied muscles were not significantly correlated with the changes in the sprint velocity with wind velocity correction at the 0–100 m interval (*p* = 0.297–0.497 and *ρ* = −0.154–0.406).

## 4 DISCUSSION

The present longitudinal study revealed that the relative volumes of TFL, SAR, BFlh, BFsh, ST, and IL significantly increased after a 1‐year observation period in well‐trained sprinters. Furthermore, among the hypertrophied muscles, only the change in the relative volume of ST was significantly positively correlated with the change in sprint velocity at the 50–60 m interval. These results suggest that ST hypertrophy seems to be associated with an improvement in sprint performance during the maximal velocity phase of the 100‐m sprint within well‐trained sprinters.

### 4.1 Changes in the sprint performance and muscle morphology

The sprint velocities at the 0–100 m, 0–10 m, and 50–60 m intervals increased after the observation period. Additionally, the step frequency increased, and the flight time decreased at 0–10 m intervals. In addition, the stance time shortened, and flight distance increased at 50–60 m intervals. These results are partially consistent with a previous longitudinal study showing decreases in the sprint times at various intervals (0–10, 40, 80, 150, and 300 m) after 6‐month daily training for sprint performance enhancement (Nuell et al., 2019). The present results indicate that the 1‐year daily training consisting of sprint running, technique drills, jumps, and resistance exercises for the trunk and thigh muscles improves the sprint performance within well‐trained sprinters.

After the observation period, the relative volumes of the six trunk and thigh muscles (TFL, SAR, BFlh, BFsh, ST, and IL) increased. These results are similar to the findings from previous cross‐sectional studies (Ema et al., 2018; Handsfield et al., 2017; Miller et al., 2021; Takahashi et al., 2021b) that the relative volumes of TFL, SAR, BFlh, BFsh, and ST were larger in well‐trained sprinters than in non‐sprinters. Meanwhile, there were no changes in the relative volumes of the eight trunk and thigh muscles (Gra, VLVI, VM, thigh ADDs, SM, *G*_max_, or PM) after the observation period in the present study, although the larger relative sizes of these muscles were observed in well‐trained sprinters than in non‐sprinters (Ema et al., 2018; Handsfield et al., 2017; Miller et al., 2021; Takahashi et al., 2021b). It was suggested that the magnitude of muscular adaptation after long‐term resistance training was smaller in well‐trained athletes than in nonathletes (Peterson et al., 2005). In our study, the athletes had a long history (7.9 ± 1.8 years) of training for improving the sprint performance at the beginning of the experiment. Thus, the athletes may have had less room for hypertrophy of some muscles in response to daily training. Understanding the underlying reasons for the lack of hypertrophy may help improve training programs aimed at enhancing sprint performance in the well‐trained sprinters.

Surprisingly, the relative volume of RF decreased after the 1‐year observation period in this study. Skeletal muscle atrophy due to an imbalance in muscle synthesis and degradation is mainly caused by two extrinsic factors: muscle inactivity and insufficient energy intake (Boirie, 2009). It has been shown that the surface electromyographic activity of RF was higher during knee extension‐only exercise than during hip flexion‐only exercise (Miyamoto et al., 2012) and multi‐joint exercise (leg press (Ema et al., 2016)). In the present study, athletes did not perform knee extension‐only exercises, but mainly performed hip flexion‐only (e.g., hip flexion with manual resistance) and multi‐joint exercises (e.g., sprint, squat, and hang clean) throughout the 1‐year daily training. Thus, RF is unlikely to be sufficiently recruited during our daily training. Meanwhile, a notable observation made earlier was that some less‐recruited arm and leg muscles were atrophied during resistance training, whereas the recruited muscles were hypertrophied, especially when energy availability was limited (Van Vossel et al., 2023). If this observation (referred to as muscle mass reallocation) is applicable to our study, the nutritional status of the athletes may be a factor for RF atrophy. Future studies are warranted to better understand the plasticity of less‐recruited muscles during daily training in terms of the daily energy intake.

### 4.2 Relationship between the changes in muscle morphology and sprint performance

Among the hypertrophied muscles (TFL, SAR, BFlh, BFsh, ST, and IL), only the change in the relative volume of ST was positively correlated with the change in sprint velocity at 50–60 m interval. Similar to our finding, a previous cross‐sectional study reported that sprinters with a large ST showed high sprint velocity during the maximal velocity phase (50–60 m interval (Takahashi et al., 2021a)). However, there has been uncertainty about whether hypertrophy of the specific muscle is linked to an improvement in the sprint performance within well‐trained sprinters owing to the multifactorial nature of sprint running. Our longitudinal study provides the finding that ST hypertrophy seems to be associated with an improvement in sprint performance during the maximal velocity phase. Meanwhile, the changes in the relative volumes of the hypertrophied muscles were not significantly correlated with either the changes in sprint velocities at the 0–100 m or 0–10 m intervals in the present study. The present results suggest that the hypertrophy of some trunk and thigh muscles does not necessarily influence sprint performance during the entire 100‐m sprint or its initial acceleration phase.

Two possible factors, neural activation and morphological properties, may explain the positive correlation between the change in the relative volume of ST and the change in sprint velocity at 50–60 m interval. The electromyographic activity of ST has been reported to be higher, especially from the middle flight to the late stance phases, in which hip extension and knee flexion torques are generated during the maximal velocity phase (Higashihara et al., 2018). In addition, ST has relatively long fibers among the hip extensor muscles (Friederich et al., 1990). Muscle fiber length is a determinant of the maximal shortening velocity of a muscle (Bodine, 1982). Theoretically, a muscle with longer fibers can generate a greater force than a muscle with shorter fibers for a given shortening velocity. Thus, ST has great potential for generating hip extension torque from the middle flight to the late stance phases during the maximal velocity phase with a substantially high angular velocity of hip extension (predicted to be > 800°/s during the stance phase (Miyashiro et al., 2019)). The hip extension velocity from the late flight phase to the early stance phase was considered a key mechanical component in reducing the horizontal braking force (Ito et al., 1998) and accelerating the subsequent leg backward swing velocity during the stance phase (Ito et al., 1998). The maximal backward leg swing velocity during the stance phase was reported to be significantly positively correlated with the maximal sprint velocity (Ito et al., 1998) through the generation of a horizontal propulsive force (Morin et al., 2015). Taken together, ST hypertrophy may contribute greatly to the generation of hip extension torque, especially during fast movements, thereby increasing the maximal sprint velocity.

In this study, the changes in the relative volumes of TFL, SAR, BFlh, BFsh, ST, and IL were not correlated with the change in sprint velocity at the 0–10 m interval. These findings are partially consistent with a previous study showing no significant correlations between the changes in the relative volumes of three muscle groups (quadriceps femoris, thigh ADDs, and hamstrings) and the change in sprint time at the 0–10 m interval (Nuell et al., 2019). The present results suggest that the hypertrophy of some trunk and thigh muscles does not greatly affect sprint performance during the initial acceleration phase. Previous studies have reported that sprint performance during the acceleration phase is more dependent on the forward orientation of the resultant ground reaction force vector applied to the supporting ground than its magnitude (Kugler et al., 2010; Morin et al., 2011). Thus, changes in the ability to orient the ground reaction force forward may be one of the factors that change sprint performance during the initial acceleration phase.

### 4.3 Practical implications

The present study provides significant practical implications for sports and clinical settings. In sports, some coaches and athletes may design resistance training programs for uniformly strengthening several muscles in each trunk and thigh muscle group (e.g., the hamstrings) to improve the sprint performance. However, hypertrophy of muscles that contribute less to sprint running can impair sprint velocity by increasing the body mass (Ropret et al., 1998). Meanwhile, our study highlights that among the hypertrophied trunk and thigh muscles, only ST hypertrophy is associated with sprint performance improvement. Thus, training focusing on ST hypertrophy may be a beneficial strategy for increasing the maximal sprint velocity. Recent studies have demonstrated that ST was preferentially hypertrophied among the hamstring muscles after the Nordic hamstring (Bourne et al., 2017) and isometric hip extension training (Carmichael et al., 2022). Additionally, ST was reported to be highly activated during the Nordic hamstring in the hip flexed position (90° of hip flexion) than during that in the hip neutral position (0° of hip flexion (Hegyi et al., 2019)). Thus, these resistance exercises may be recommended to be incorporated into daily training programs to enhance sprint performance.

Based on the cross‐sectional findings of significant associations between the sizes of some muscles (e.g., SAR and ST) and sprint performance (Miller et al., 2022; Takahashi et al., 2021a), some practitioners may focus on the hypertrophy of specific muscles to enhance sprint performance during their daily training. Meanwhile, our longitudinal study revealed that the hypertrophy of some trunk and thigh muscles (e.g., SAR) induced by daily training was not significantly associated with an improvement in sprint performance. Thus, the findings from cross‐sectional studies may not be directly applicable in designing training programs for sprint performance improvement within well‐trained sprinters. These suggestions underscore the importance of transitioning from cross‐sectional to longitudinal studies to gain deeper insights into achieving high sprint velocity. Meanwhile, the present results only apply to well‐trained sprinters who performed daily training for a relatively short period (1 year). Hence, future studies are warranted to examine whether our results hold in other populations (e.g., untrained population) and longer training periods (more than 1 year) to better understand the detailed relationship between muscle morphological changes and changes in sprint performance.

### 4.4 Limitations and future directions

This study has several limitations that should be addressed. First, the sprint performance of each athlete was measured only 1 day before and after the observation period due to the athletes' limited schedule, although there is a day‐to‐day variability in sprint performance. However, 15 out of the 23 athletes competed in an official 100‐m race during the experimental period (November 2021 to November 2022), and the 13 athletes broke their own season's best record corrected for wind velocity. Additionally, the corrected 100‐m season's best record was significantly shorter in the 2022‐year season (11.42 s [10.93–11.77 s]) than in the 2021‐year season (11.43 s [10.99–11.80 s] and [*p* = 0.041 and *r* = 0.37]). Thus, the present results of changes in sprint performance would reflect the improvements in the athletes' sprint performance. Second, wind velocities during sprints were not comparable before and after the 1‐year observation period. Although the velocities of 100‐m sprints were corrected for wind velocity using the previous equation (Moinat et al., 2018), no equations have yet been established to correct the sprint velocity at each interval for wind velocity. In the present study, the change in mean corrected 100‐m sprint velocity before and after the observation period (0.17 m∙s^−1^) is 85% of the change in mean 100‐m sprint velocity (0.20 m∙s^−1^ [Supporting Information S2]). This implies that the effect of wind velocity on the 100‐m sprint velocity was <20%. Although the changes in the wind velocity between measurements during sprints may potentially mask the relationship between changes in sprint performance and changes in muscle morphology, they may not have a significant effect on the underlying changes in 100‐m sprint performance. Third, the cameras at frame rates of 240 fps and 250 fps were used in the present study, and thus they could not detect small changes of <0.004 s in some spatiotemporal variables (e.g., stance and flight times) of each athlete before and after the observation period. Future studies should use cameras with a higher time resolution to better understand the daily training‐induced changes in spatiotemporal variables. Fourth, the present study did not focus on examining the changes in relevant factors (e.g., neurophysiological and histochemical factors (Häkkinen et al., 1998)), other than muscle size, for sprint performance. Additionally, we only examined the correlation between changes in muscle volume and changes in sprint performance using the Spearman's rank correlation test, which does not allow us to adjust for potential confounders. Future work may indicate the extent to which muscle hypertrophy, especially in ST, affects sprint performance when compared to the changes in other factors. Next, some participants wore different sprint spikes in the sprint performance measurements before and after the observation period. However, studies reported that the sprint velocity or time did not significantly change in conditions with different spike bending stiffness when compared to the control condition (Nagahara et al., 2018; Smith et al., 2016). Thus, the use of different sprint spikes would not have a substantial effect on changes in sprint performance before and after the observation period. Finally, this study was an observational study on male collegiate sprinters. Thus, our results may not be directly applicable to other athletic teams with different training regimens or other populations (e.g., female sprinters and athletes in other sports events), which is an important future theme.

## 5 CONCLUSION

The present longitudinal study was designed to address an unanswered question of cross‐sectional studies: whether hypertrophy of the individual trunk and thigh muscles induced by daily training (e.g., sprint, jump, and resistance training) is linked to an improvement in sprint performance within well‐trained sprinters. The results showed that the relative volumes of TFL, SAR, BFlh, BFsh, ST, and IL significantly increased after a 1‐year observation period in well‐trained sprinters. Furthermore, among the hypertrophied muscles, only the change in the relative volume of ST was significantly positively correlated with the change in sprint velocity at the 50–60 m interval. This is the first longitudinal study to suggest that ST hypertrophy seems to be associated with an improvement in the sprint performance within well‐trained sprinters during the maximal velocity phase.

## CONFLICT OF INTEREST STATEMENT

The authors declare that they have no conflicts of interest that are relevant to the content of this article.

## CONSENT TO PARTICIPATE

All participants completed an informed consent form after a careful explanation of the purpose, procedures, and potential risks of this study.

## Supporting information

Supporting Information S1

Supporting Information S2

Supporting Information S3

Supporting Information S4

## Data Availability

The data sets generated and analyzed during the current study are available from the corresponding author on reasonable request.
