# Supplementary material for: Muscle morphological changes and enhanced sprint running performance: A 1‐year observational study of well‐trained sprinters
Source: Eur J Sport Sci. 2024 Jun 21;24(9):1228–39. doi: 10.1002/ejsc.12155 (PMC11369333; doi:10.1002/ejsc.12155)
Supplement: Supplementary file 1 — Supporting Information S1 [file EJSC-24-1228-s003.docx]

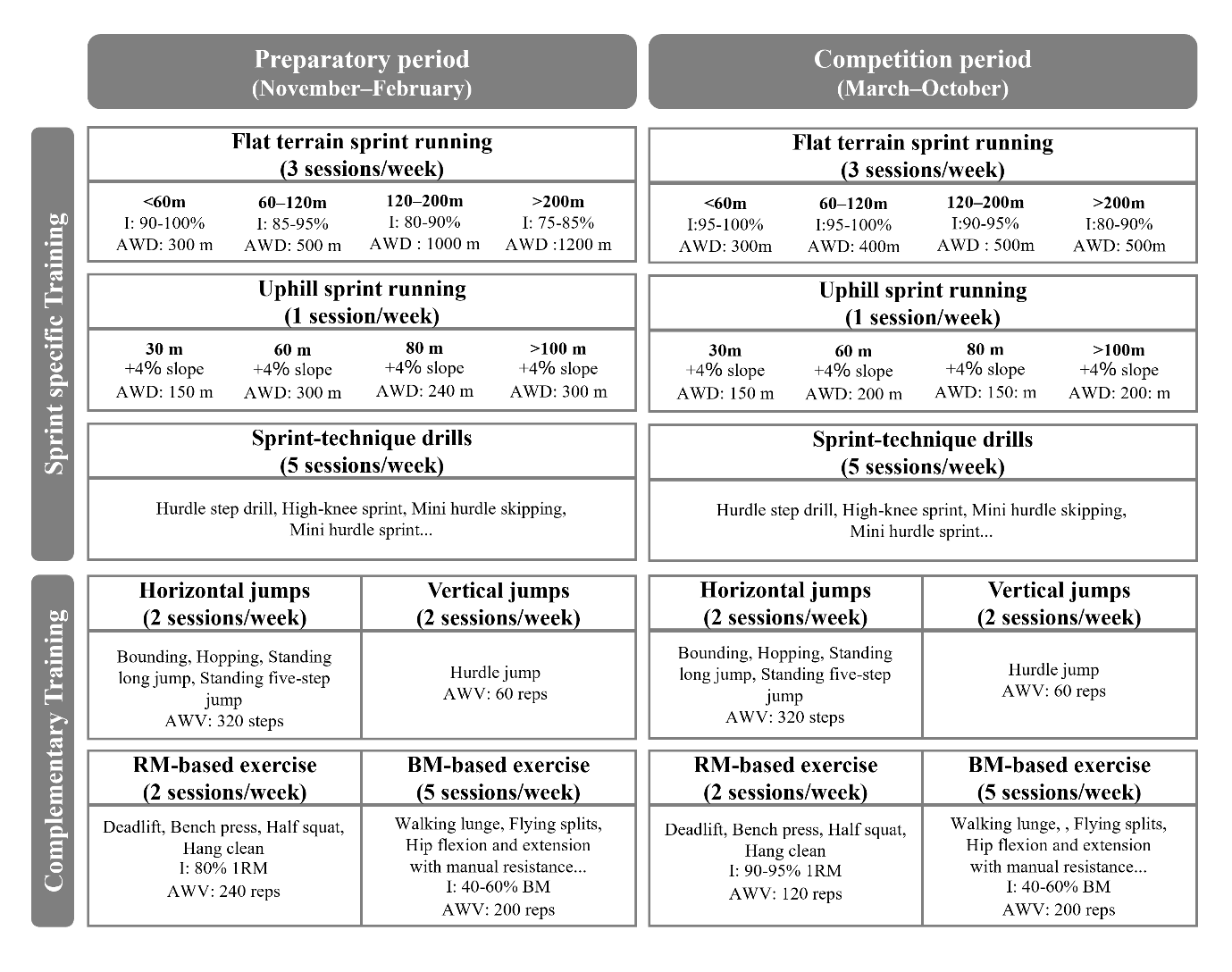
**Supplemental content 1. Overview of the content of daily training for improving sprint performance.** I: intensity, AWD: average weekly distance, AWV: average weekly volume, RM: repetition maximum, BM: body mass.
