# Supplementary material for: Muscle morphological changes and enhanced sprint running performance: A 1‐year observational study of well‐trained sprinters
Source: Eur J Sport Sci. 2024 Jun 21;24(9):1228–39. doi: 10.1002/ejsc.12155 (PMC11369333; doi:10.1002/ejsc.12155)
Supplement: Supplementary file 2 — Supporting Information S2 [file EJSC-24-1228-s001.docx]

**Supplemental content 2**

**Changes in the sprint performance variables after the one-year observation period.**

| **Interval** | **Sprint performance variable** | **Before** | |  | **After** | |  | | **%Change** | |  |
| --- | --- | --- | --- | --- | --- | --- | --- | --- | --- | --- | --- |
| **100 m** | Time (s) | 11.70 | ± | 0.40 | 11.45 | ± | | 0.44 | −2.2 | ± | 2.4 |
|  | Velocity (m∙s^−1^) | 8.55 | ± | 0.29 | 8.75 | ± | | 0.34 | 2.3 | ± | 2.5 |
|  | Corrected velocity (m∙s^−1^) | 8.52 | ± | 0.29 | 8.69 | ± | | 0.33 | 2.0 | ± | 2.3 |
| **0–10 m** | Velocity (m∙s^−1^) | 5.39 | ± | 0.18 | 5.48 | ± | | 0.20 | 1.8 | ± | 3.2 |
|  | Step frequency (Hz) | 4.542 | ± | 0.371 | 4.687 | ± | | 0.340 | 3.0 | ± | 5.7 |
|  | Step length (m) | 1.546 | ± | 0.146 | 1.533 | ± | | 0.125 | −0.9 | ± | 5.4 |
|  | Flight time (s) | 0.096 | ± | 0.018 | 0.088 | ± | | 0.015 | −9.7 | ± | 17.7 |
|  | Stance time (s) | 0.126 | ± | 0.014 | 0.126 | ± | | 0.013 | −0.1 | ± | 11.4 |
|  | Flight distance (m) | 0.667 | ± | 0.128 | 0.644 | ± | | 0.112 | −6.0 | ± | 15.9 |
|  | Stance distance (m) | 0.869 | ± | 0.065 | 0.889 | ± | | 0.088 | 1.7 | ± | 7.5 |
| **50–60 m** | Velocity (m∙s^−1^) | 9.51 | ± | 0.37 | 9.71 | ± | | 0.43 | 2.1 | ± | 3.5 |
|  | Step frequency (Hz) | 4.587 | ± | 0.283 | 4.606 | ± | | 0.227 | 0.4 | ± | 4.3 |
|  | Step length (m) | 2.088 | ± | 0.121 | 2.117 | ± | | 0.123 | 1.3 | ± | 3.1 |
|  | Flight time (s) | 0.123 | ± | 0.012 | 0.124 | ± | | 0.009 | 0.9 | ± | 6.2 |
|  | Stance time (s) | 0.096 | ± | 0.006 | 0.092 | ± | | 0.006 | −4.0 | ± | 4.7 |
|  | Flight distance (m) | 1.139 | ± | 0.112 | 1.180 | ± | | 0.088 | 3.5 | ± | 6.0 |
|  | Stance distance (m) | 0.949 | ± | 0.042 | 0.936 | ± | | 0.061 | −1.6 | ± | 5.3 |

The data are presented as mean ± standard deviation.
