# Supplementary material for: Muscle morphological changes and enhanced sprint running performance: A 1‐year observational study of well‐trained sprinters
Source: Eur J Sport Sci. 2024 Jun 21;24(9):1228–39. doi: 10.1002/ejsc.12155 (PMC11369333; doi:10.1002/ejsc.12155)
Supplement: Supplementary file 3 — Supporting Information S3 [file EJSC-24-1228-s002.docx]

# Supplemental content 3

**Changes in the absolute and relative volumes of 14 trunk and thigh muscles after the one-year observation period.**

| **Muscle** | **Absolute volume (cm^3^)** | | | |  | **Relative volume (cm^3^∙kg^−1^)** | | | |
| --- | --- | --- | --- | --- | --- | --- | --- | --- | --- |
|  | **Before** | | **After** | |  | **Before** | | **After** | |
| **TFL** | 77 | 64 to 94 | 85 | 67 to 93 |  | **1.22** | 0.98 to 1.42 | **1.28** | 1.03 to 1.41***** |
| **SAR** | **158** | 134 to 191 | **162** | 141 to 193***** |  | **2.52** | 2.09 to 2.68 | **2.47** | 2.27 to 2.66***** |
| Gra | 129 | 116 to 151 | 129 | 112 to 148 |  | 1.94 | 1.77 to 2.26 | 1.88 | 1.69 to 2.25 |
| **RF** | **317** | 288 to 355 | **312** | 279 to 326***** |  | **4.98** | 4.42 to 5.25 | **4.67** | 4.22 to 5.00****** |
| VLVI | 1277 | 1116 to 1372 | 1287 | 1121 to 1356 |  | 18.98 | 18.15 to 19.73 | 19.20 | 18.49 to 19.86 |
| VM | 461 | 416 to 503 | 461 | 425 to 506 |  | 6.97 | 6.68 to 7.57 | 7.02 | 6.62 to 7.51 |
| ADDs | 1161 | 1059 to 1236 | 1181 | 1048 to 1233 |  | 17.58 | 16.90 to 18.27 | 17.61 | 16.71 to 18.40 |
| **BFlh** | **239** | 209 to 270 | **247** | 227 to 279****** |  | **3.62** | 3.29 to 3.93 | **3.89** | 3.47 to 4.08****** |
| **BFsh** | **100** | 85 to 111 | **112** | 101 to 125****** |  | **1.53** | 1.32 to 1.64 | **1.69** | 1.58 to 1.86****** |
| **ST** | **279** | 259 to 310 | **286** | 270 to 313****** |  | **4.24** | 3.93 to 4.50 | **4.35** | 4.09 to 4.70****** |
| SM | 305 | 267 to 326 | 300 | 276 to 314 |  | 4.55 | 4.18 to 4.97 | 4.47 | 4.00 to 4.72 |
| Gmax | 1034 | 973 to 1159 | 1052 | 966 to 1179 |  | 16.16 | 15.68 to 17.45 | 16.01 | 15.23 to 17.70 |
| PM | 308 | 254 to 354 | 291 | 261 to 348 |  | 4.65 | 4.15 to 5.00 | 4.46 | 4.15 to 5.18 |
| **IL** | **212** | 188 to 225 | **227** | 207 to 241****** |  | **3.21** | 2.98 to 3.45 | **3.54** | 3.14 to 3.83****** |

The data are presented as median values (interquartile ranges). Significant difference before and after the one-year observation period: **p* < 0.05 and ***p* < 0.01. Measured variables with significant changes are emboldened. TFL: Tensor fasciae latae, SAR: Sartorius, Gra: Gracilis, RF: Rectus femoris, VLVI: Vastus lateralis and intermedius, VM: Vastus medialis, ADDs: Adductors, BFlh: Biceps femoris long head, BFsh: Biceps femoris short head, ST: Semitendinosus, SM: Semimembranosus, Gmax: Gluteus maximus, PM: Psoas major, IL: Iliacus.

^a^As for SAR, the relative volume (the median value) after the observation period (2.47 cm^3^∙kg^−1^) was lower than that before the period (2.52 cm^3^∙kg^−1^), although the percent change (median value) in the relative volume before and after the observation period was presented as positive value (2.9% [Fig. 3]). The Wilcoxon signed-rank test showed that the sum of ranks for the positive changes in the relative volume of SAR was 251, whereas the sum of ranks for its negative changes was 61. These results imply an increase in the relative volume of SAR after the observation period.
