# Supplementary material for: Muscle morphological changes and enhanced sprint running performance: A 1‐year observational study of well‐trained sprinters
Source: Eur J Sport Sci. 2024 Jun 21;24(9):1228–39. doi: 10.1002/ejsc.12155 (PMC11369333; doi:10.1002/ejsc.12155)
Supplement: Supplementary file 4 — Supporting Information S4 [file EJSC-24-1228-s004.docx]

# Supplemental content 4

**Rank correlation coefficient values (*ρ*) between the changes in the relative volumes of the hypertrophied muscles and the change in the sprint velocity at each interval after the one-year observation period.**

| **Variable** | **Change in the sprint velocity at each interval (m∙s^−1^)** | | | |
| --- | --- | --- | --- | --- |
|  | **100 m** | **100 m (corrected)** | **0–10 m** | **50–60 m** |
| **Changes in the relative volumes (cm^3^∙kg^−1^)** |  |  |  |  |
| TFL | 0.148 | 0.149 | 0.054 | 0.133 |
| SAR | −0.133 | −0.154 | 0.023 | −0.159 |
| BFlh | 0.176 | 0.203 | 0.396 | 0.169 |
| BFsh | 0.322 | 0.353 | 0.263 | 0.199 |
| **ST** | 0.433 | 0.406 | 0.510 | **0.591*** |
| IL | 0.257 | 0.277 | 0.163 | 0.361 |

Significant correlation between both variables: *Corrected *p* < 0.05. Measured variable with a significant correlation is emboldened. TFL: Tensor fasciae latae, SAR: Sartorius, BFlh: Biceps femoris long head, BFsh: Biceps femoris short head, ST: Semitendinosus, IL: Iliacus.
